# The diagnostic value of LncRNA NEAT1 targeting miR-129-5p in pancreatic cancer patients

**DOI:** 10.1038/s41598-025-12963-y

**Published:** 2025-07-29

**Authors:** Hadeer Saied Mahmoud, Noha Abdel-Rahman Eldesoky, Olfat G. Shaker, Ahmed A. El-Husseiny

**Affiliations:** 1 Department of Biochemistry, Faculty of Pharmacy, Egyptian Russian University, Badr City, Cairo, 11829 Egypt; 2 Biochemistry and Molecular Biology Department, Faculty of Pharmacy (Boys and Girls), Al-Azhar University, Nasr City, Cairo Egypt; 3 Department of Medical Biochemistry and Molecular Biology, Faculty of Medicine, Cairo University, Cairo, 11562 Egypt

**Keywords:** LncRNA, NEAT1, Pancreatic cancer, Metastasis, miR-129-5p, Cancer, Biomarkers, Oncology

## Abstract

Early pancreatic cancer (PC) identification and management has gained much clinical and research attention. Recent reports have demonstrated the important function of circulating noncoding RNAs (ncRNAs) in the diagnosis and prognosis of malignancies. However, the clinical value of serum ncRNAs in PC has not been fully clarified. Hence, we investigated serum levels of long-ncRNA NEAT1 and miR-129-5p in PC cases, exploring their relationship with related targets BCL2 and TGF-β1. Serum NEAT1 and miR-129-5p levels were evaluated in 60 treatment-naïve PC cases and 30 apparent healthy individuals by RT-PCR. Besides, serum TGF-β1 and BCL2 levels were measured by ELISA, whereas CA19-9 and CEA were measured utilizing the chemiluminescence technique. We demonstrated that serum NEAT1, BCL2, and TGF-β1 levels were significantly upregulated, whereas serum levels of miR-129-5p were markedly reduced in PC cases compared to controls. miR-129-5p had a higher diagnostic value for PC than NEAT1, with specificity, sensitivity, and AUC of 100%, 95%, and 0.96 versus 93.3%, 83.3%, and 0.89, respectively. Moreover, miR-129-5p had a higher AUC than CA19-9 and CEA. Additionally, serum miR-129-5p was significantly downregulated in PC cases with T3 or T4 stages compared to those with T2 stage & negatively correlated with NEAT1, BCL2, and TGF-β1, whereas NEAT1 was notably positively correlated with BCL2 and TGF-β1. Collectively, serum NEAT1 and miR-129-5p could be beneficial biomarkers for the detection of PC. Also, our findings accentuate the correlation between NEAT1, miR-129-5p, BCL2, and TGF-β1 and provide PC therapeutic targets.

**Supplementary Information:**

The online version contains supplementary material available at 10.1038/s41598-025-12963-y.

## Introduction

Pancreatic cancer (PC) ranks tenth concerning cancer incidence and the fourth predominant cause of mortality owing to its poor survival rate^1^. Globally in 2020, there were 466,003 fatalities and 495,773 new patients of PC^2^. A crucial and challenging task for PC has always been early identification, diagnosis, and treatment. Therefore, clarifying the fundamental mechanisms of its development and creating innovative methods with increased diagnostic efficacy are urgently needed^3^. PC is an inherited condition brought on by various environmental variables and complicated interactions within the tumor microenvironment^4^.

Long noncoding RNAs (lncRNAs) are ncRNAs that surpass 200 nucleotides and are incapable of encoding proteins^5^. Lately, it has been established that lncRNAs take part in a diversity of signaling pathways. LncRNAs target distinct chromatin modification complexes to control the activation or inactivation of genes^6^. In order to interfere with the regulation of genes, lncRNAs can potentially interact with mRNA-stabilizing proteins or enlist microRNAs (miRNAs) as scaffolds^7^. Human tumors have been shown to exhibit dysregulation of lncRNAs. Notably, The function of lncRNAs as tumor inhibitors or oncogenes is to modulate the growth, invasion, migration, differentiation, and autophagy of cancer cells^8^. Growing data suggests that we can use lncRNAs as indicators for the prognosis and diagnosis of tumors^9,10^.

Interestingly, nuclear-enriched abundant transcript 1 (NEAT1) is a recently identified crucial element of nuclear paraspeckles that is transcribed from human chromosome 11q13.1^11^. In plenty of human cancers kinds, like hepatocellular carcinoma, melanoma, colon, breast, and pancreatic cancers, NEAT1 serves as an oncogenic lncRNA^12^. In order to control oncogenic factors in cancer, NEAT1 competitively binds to a range of miRNAs that have regulatory functions in apoptosis, proliferation, metastasis, invasion, and epithelial-mesenchymal transition (EMT)^13^. Besides, NEAT1 can epigenetically modulate genes expression. It has been proved that NEAT1 increases the miR-129 gene’s DNA methylation, which inhibits the tumor suppressor miR-129-5p’s expression in breast cancer^14^. Moreover, NEAT1 enhances the resistance to histone deacetylase inhibitors in nasopharyngeal carcinoma by repressing miR-129^15^.

It is believed that miR-129-5p’s downstream targets are anti-apoptotic B-cell lymphoma 2 (BCL2)^16^ and transforming growth factor beta (TGF-β)^17–19^. Numerous pathways leading to EMT-specific cellular alterations can be activated by TGF-β^20^. Therefore, this study was conducted to evaluate the serum expression levels of lncRNA NEAT1/miR-129-5p and their related targets BCL2 and TGF-β1 in PC patients. Besides, their diagnostic efficacy for PC and their association with PC clinicopathologic features were evaluated.

## Patients and methods

### Study participants

In the present study, ninety adult participants were divided into 60 PC cases (mostly adenocarcinomas) and 30 apparently healthy controls. The age groupings of PC cases and controls were as nearly matched as feasible. All participants were recruited from the Gastrointestinal Endoscopy Unit, Kasr Al-Ainy Hospital, Cairo University between April and August 2024. The endoscopic ultrasound and histopathology findings verified the PC diagnosis. Each participant’s full medical history, physical examination, serum creatinine, fasting plasma glucose (FPG), liver function tests, and complete blood count were carefully recorded upon registration.

Medical records were used to collect and document the clinicopathological characteristics of PC patients, including tumor size, the quantity of positive lymph nodes (LNs), and distant metastases. The patients’ age mean ± standard deviation (SD) was 55.1 ± 9.28 years. The eighth edition of the tumor-node-metastasis (TNM) staging approach established by the American Joint Committee on Cancer (AJCC) was used to assess the PC stages^21^. Patients with different cancer stages were then subclassified principally into two categories: metastatic (stage IV) and curative (stages II, and III). Every PC patient with stage IV was diagnosed initially as metastatic. Histologic cancer grading was determined according to Hruban and Fukushima, (2007)^22^.

This study was approved by the ethics committee of the Cairo University’s Faculty of Pharmacy’s with approval number (BC3568), all methods were performed in accordance with relevant guidelines and regulations, and the written informed consent was obtained from all subjects in advance. The study has been conducted in accordance with the Declaration of Helsinki. Adult patients (over the age of 18 years) of both genders with a recently diagnosed confirmed PC met the inclusion criteria. Individuals who have previously received PC treatment, any cancer other than PC, or pancreatitis were excluded. Additionally, those with a history of alcohol use and smokers were not included.

### Sampling

Six ml of each participant’s venous blood were drawn and gathered for serum separation into yellow gel vacutainers. After 30 min of coagulation, the blood was centrifuged for 10 min at 4000 rpm. Subsequent separated sera aliquots were maintained in the Molecular Biology lab at −80 °C, Faculty of Medicine, Cairo University until the time of RNA extraction as well as the assessment for BCL2 and TGF-β1 levels.

### Selection of NEAT1/miR-129-5p/target genes (BCL2 and TGF-β1) coexpression networks

According to the LncRNA and Disease Database v3.0 (http://www.rnanut.net/lncrnadisease/), NEAT1 was the top causative ncRNA with compelling evidence of being linked to pancreatic ductal adenocarcinoma with relationship score of 0.985791 that has been empirically verified. Also, NEAT1 was selected based on experimental evidence of its mechanistic significant association with PC in both tumour tissues and cell lines^12,23–25^. The miRNA targets of NEAT1 were then obtained by accessing the transcriptome-wide miRNA target predictions from the miRcode 11 database (http://www.mircode.org). It is noteworthy that this database indicated that the miR-129-5p/129ab-5p family was the highly conserved target of NEAT1. Besides, the ENCORI/starBase platform was employed to forecast the miR-129-5p and NEAT1 binding locations. Moreover, it has been established experimentally in earlier studies that NEAT1 can interact with miR-129-5p^26,27^. The Human MicroRNA Disease Database v4 indicated the relation between miR-129-5p and pancreatic carcinoma. Using the TargetScan Human 7.2 database, it was predicted that BCL2 would be a direct target for miR-129-5p. Also, this relationship was experimentally verified in cancer cell lines. Additionally, miR-129-5p targets several genes related to angiogenesis that resulted in the TGF-β1 upregulation indirectly in several cancer cell lines^17,18^.

## Methodology

### Total RNA extraction and purity assessment

Total RNA extraction was conducted utilizing the miRNeasy Mini kit (Qiagen, Valencia, CA, USA) in compliance with the guidelines of the manufacturer. After extraction, the RNA was maintained in aliquots at −80 °C after being eluted in 50 µl of Rnase-free water. For assessing RNA concentration and purity, the spectrophotometer used was the NanoDrop^®^− 1000 (NanoDrop Technologies, Inc., Wilmington, USA).

The ratios of 260/280 and 260/230 of at least 1.9 were considered acceptable.

### Reverse transcription

Reverse transcription was accomplished using the miScript II RT kit (Qiagen, Valencia, CA, USA) on RNA (60 ng) in an ultimate volume of 20 uL RT reactions complying with the directions provided by the manufacturer. The miScript reverse transcriptase was inactivated by incubating it for 60 min at 37 °C and 5 min at 95 °C.

### Assessment of NEAT1 and miR-129-5p gene expression

NEAT1 and miR-129-5p expression levels were measured by qPCR employing the miScript SYBR Green PCR kit from Qiagen. For normalizing NEAT1 and miR-129-5p expressions, glyceraldehyde-3-phosphate dehydrogenase (GAPDH) and SNORD68 were employed as references, respectively. It has previously been confirmed that the GAPDH gene is a great internal control for lncRNA normalization. Notably, GAPDH and SNORD68 have been thoroughly validated in many investigations as a trustworthy internal control for lncRNA and miRNA normalization, respectively, exhibiting consistent and stable expression in serum samples from both healthy controls and PC patients^28–32^. This validation strengthens their utility as a reference gene for lncRNA and miRNA relative quantification, respectively. Additionally, we compared the serum expression levels of housekeeping genes (GAPDH and SNORD68) between controls and PC patients sample sets and there was no notable difference being observed regarding Cq values between the 2 groups, indicating that GAPDH and SNORD68 were stably expressed and appropriate reference genes. Primers for GAPDH (Catalog no. 330701 LPH31725A, Accession no. ENST00000496049.0) and NEAT1 (Catalog no. 330701 LPH15809A, Accession no. NR_028272.1) were supplied by Qiagen, Valencia, CA, USA. Additionally, Qiagen, Germany, provided the primers for SNORD 68 (Catalog no: MS00033712) and miR-129-5p (Catalog no: MS00006643). The PCR cycling methodology for NEAT1 and miR-129-5p assessment in total reaction volumes of 25 µL and 20 µL, respectively, was accomplished employing the Rotor-gene Q real-time PCR system (Qiagen, USA): 40 cycles at 94 °C for 15 s, 55 °C for 30 s, and 70 °C for 30 s, after proceeding 15 min at 95 °C. For the estimation of Ct values, the procedures were conducted in triplicate for each independent RNA sample, and the mean results were then computed. Melting curve investigation validated the PCR fragments’ specificity. Finally, the 2^−ΔΔCt^ method was employed to determine the relative expression for NEAT1 and miR-129-5p.

### Determination of serum BCL2 and TGF-β1 using ELISA

Serum BCL2 and TGF-β1 were investigated using a sandwich enzyme linked immunosorbent assay (ELISA) employing the Human BCL2 (Catalog no: E-EL-H0114) and TGF-β1 (Catalog no: E-EL-H0110) ELISA kits from Elabscience^®^, Texas, USA, according to the manufacturer’s directions. Positive and negative controls were included in every experiment, and every sample was investigated in duplicate, and a difference in the repeatability was less than 10%. Average the duplicate readings for each standard and samples were estimated and then subtracted the average zero standard optical density. The optical density was assessed employing a microplate reader (Stat Fax^®^ 2100, Awareness Technology, USA) set at 450 nm. Regarding precision, the inter-assay coefficient of variability (CV) was < 10% and the intra-assay CV was < 8%. A four-parameter logistic standard curves on the log-log axis were constructed, with standard concentration on the x-axis and estimated optical density values on the y-axis. By extrapolating to the standard curves (**Suppl. Figures 1 and 2**), the unknown quantity in the samples was ascertained. In advance, the concentration range of samples was predicted, and the dilution ratio was determined through a preliminary experiment. A 5-fold and 300-fold dilutions were performed for serum BCL2 and TGF-β1 determination, respectively. Finally, the actual concentration was the calculated concentration multiplied by the dilution factor.

### Determination of serum CEA and CA19-9

Serum carcinoembryonic antigen (CEA) and cancer antigen 19–9 (CA 19–9) levels were measured utilizing the chemiluminescence technique by the Access 2 Immunoassay System (Beckman Coulter, Brea, CA, USA) with detection limits of 0.8–1000 U/ml for CA 19−9 and 0.1–1000 ng/ml for CEA. Dilutions were conducted when needed for samples where the detection limits were exceeded.

### Sample size Estimation

Based on Zhao et al. study^33^, the area under the curve (AUC) for serum TGF-β1 as a diagnostic marker for PC was 0.794 and the ratio of controls to PC cases was 0.35, so the final sample size of 90 subjects (60 PC patients and 30 healthy controls) was able to reject the null hypothesis and detect power of 99.95% and type I error probability of 0.05. The sample size was estimated utilizing MedCalc statistical package version 18.2.1.

### Statistical methods

The data was managed and analyzed conducting Graph pad prism (Inc, USA) version 9. Presenting qualitative data was done using percentage and frequency whereas numerical data was summarized using means ± SD and medians (range). Qualitative variables were compared employing the Chi-square test. The Kolmogorov-Smirnov and Shapiro-Wilk tests were employed to examine the variable’s normality. Additionally, for regularly distributed numeric data, the student’s t-test was conducted whereas for non-normally distributed numeric variables, the Mann-Whitney test was utilized for contrasting between 2 groups. Notably, Kruskal-Wallis was employed to examine the variations between more than two groups, and if required, post hoc analysis (Dunn’s test) was then performed, and *P*-value was adjusted utilizing Bonferroni adjustment. Using Spearman correlation coefficients, the significance level of the association between non-normally distributed measures was assessed. The receiver operating characteristic (ROC) curve was implemented to figure out the optimal cut-off points for the serum levels of the studied variables, the AUC, ultimately sensitivity and specificity. The optimal cut-off values were selected by maximizing both specificity and sensitivity to the point where they are equal, or nearly equal as possible for the available data. Lastly, a *p*-value < 0.05 was adopted for statistical significance level. Our work complies with the Reporting Recommendations for Tumor MARKer Prognostic Studies (REMARK) prerequisites^34^.

## Results

### The demographic, laboratory, and clinicopathological findings of the studied participants

The age and gender of the PC patients and control participants were matched. Notable reductions in hemoglobin and serum albumin concentrations were observed among the PC group in comparison to control subjects. On the other hand, PC cases had notably higher serum ALT, AST, ALP, total bilirubin, FPG levels, and international normalization ratio (INR) than controls. Furthermore, there were no appreciable variations in TLC, platelet count, or serum creatinine level among the groups under study **(**Table 1**)**. The clinicopathological traits of PC cases are listed in Table 2.

**Table 1:** Demographic information and laboratory findings of PC cases and controls.

Demographic data	PC cases (*n* = 60)	Controls (*n* = 30)	*P*
Age at diagnosis (years)	55.1 ± 9.28	58.9 ± 8.86	0.07
Gender			
Males (n/%)	27 (45%)	13 (43.3%)	0.9
Females (n/%)	33 (55%)	17 (56.7%)	
Hemoglobin (g/dl)	11.4 ± 1.6	12.5 ± 1.3	0.001*
Platelet count × 10^3^/mm^3^	270.2 ± 94.7	296.7 ± 61.9	0.12
TLC (cell/mm^3^)	7.9 ± 1.8	8.4 ± 2.7	0.31
INR	1.1 ± 0.4	1 ± 0.03	0.012*
FPG (mg/dl)	125.9 ± 58.5	72.1 ± 3.9	< 0.001*
Creatinine (mg/dl)	0.8 ± 0.3	0.7 ± 0.1	0.07
Liver function tests			
ALT (IU/L)	47 (11–547)	12 (6–25)	< 0.001*
AST (IU/L)	41.5 (14–400)	14 (9–33)	< 0.001*
ALP (ng/ml)	93 (31-1416)	72 (48–115)	< 0.001*
Total bilirubin (mg/dl)	2.7 (0.4–16)	0.5 (0.4–0.7)	< 0.001*
Albumin (g/dl)	3.4 ± 0.5	4.6 ± 0.3	< 0.001*

ALT: Alanine transaminase, ALP: Alkaline phosphatase, FPG: Fasting plasma glucose, AST: Aspartate transaminase, INR: International normalization ratio, TLC: Total leucocyte count. Data are expressed as median (range) [for non-parametric data] or mean ± SD [for parametric data], and number (percentage) [for categorical data]. *: Data was taken as statistically significant at *P* < 0.05.

**Table 2:** Clinicopathological features of pancreatic cancer cases.

Clinicopathologic features		PC patients *n* (%)
**pT stage**	T2	25 (41.7)
	T3	14 (23.3)
	T4	21 (35)
**pN stage**	N1	14 (23.3)
	N2	46 (76.7)
**TNM staging**	Stage II	14 (23.3)
	Stage III	34 (56.7)
	Stage IV	12 (20)
**TNM staging groups**	Curative ( II & III)	48 (80)
	Metastatic (IV)	12 (20)
**Tumor grading**	G2	45 (75)
	G3	15 (25)

G: Tumour grading; pT: Pathological tumor; TNM: Tumor-node-metastasis; pN: pathological node; Data are expressed as n (percentage).

### Expression pattern of serum NEAT1/miR-129-5p, related targets BCL2 and TGF-β1 in PC cases and controls

**Fig. 1:**
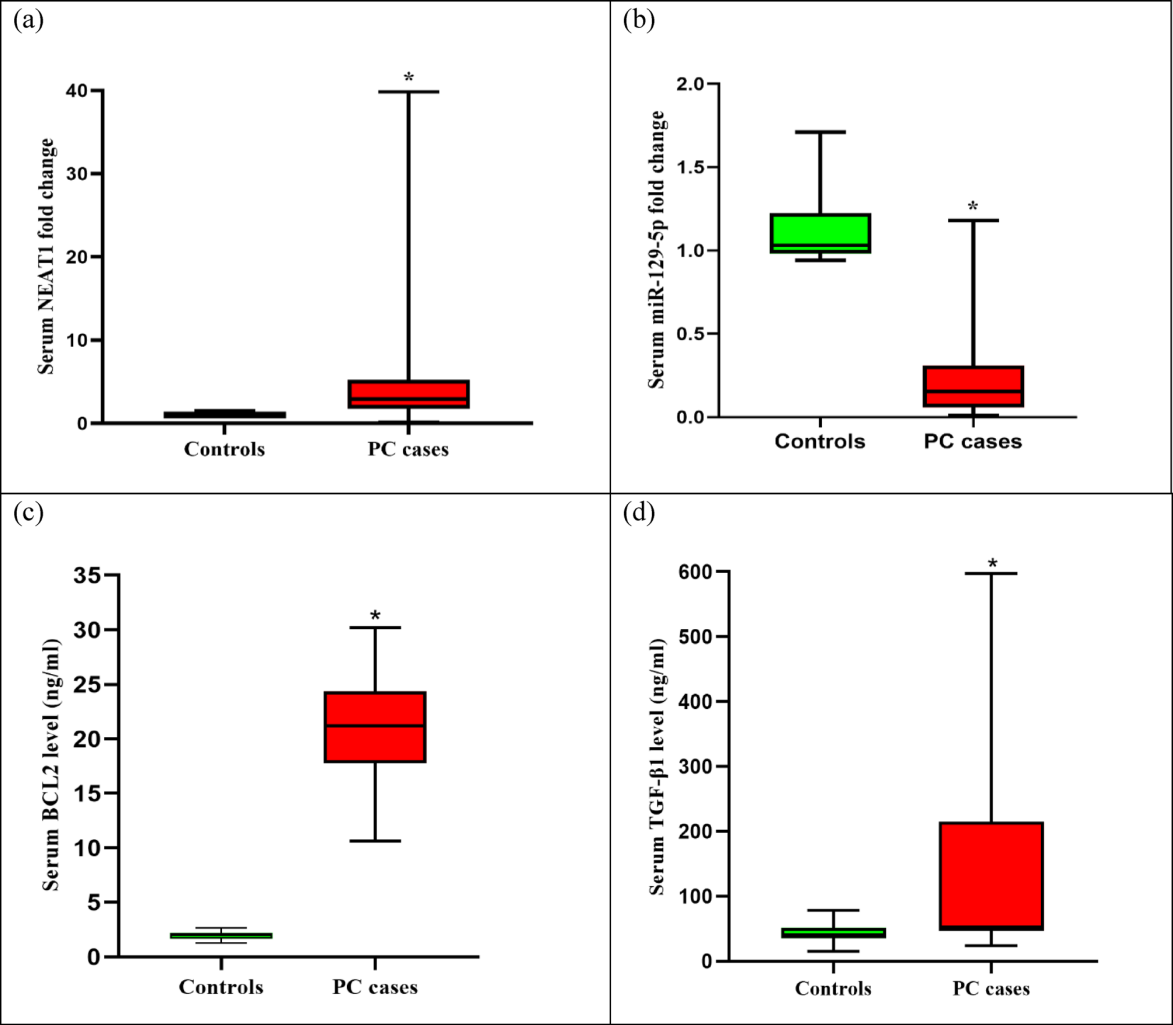
The box plots of serum NEAT1 and miR-129-5p fold changes, BCL2, and TGF-β1 protein levels in control subjects and PC cases. BCL2: B-cell lymphoma-2; NEAT1: Nuclear paraspeckle assembly transcript 1; TGF-β1: Transforming growth factor beta-1; PC: Pancreatic cancer. *: *P* < 0.05 indicating statistical significance.

Serum miR-129-5p expression was downregulated in PC patients compared to control participants with median fold change 0.16 (*P* < 0.00001; Fig. 1b), whereas serum lncRNA NEAT1 was elevated in PC patients with median fold change 2.94 (*P* < 0.00001; Fig. 1a**).** PC patients had significantly higher levels of serum BCL2 (*P* < 0.00001; Fig. 1c**)**, TGF-β1 (*P* = 0.00002; Fig. 1d**)**, CA19-9 (*P* < 0.00001), and CEA (*P* < 0.00001) than control subjects **(**Table 3**)**.

**Table 3:** Expression pattern of serum NEAT1/miR-129-5p/BCL2 and TGF-β1 in PC cases and controls.

Variable	PC cases	Controls	*P*
NEAT1 (fold change)	2.94 (0.14–39.8)	0.98 (0.84–1.52)	< 0.00001*
miR-129-5p (fold change)	0.16 (0.01–1.18)	1.03 (0.94–1.71)	< 0.00001*
BCL2 (ng/ml)	21.2 (10.6–30.2)	2 (1.3–2.7)	< 0.00001*
TGF-β1 (ng/ml)	52 (24-597.5)	40.5 (15-78.5)	0.00002*
CA19-9 (U/ml)	172 (1.3–3862)	2 (1–35)	< 0.00001*
CEA (ng/ml)	2.4 (0.8–6.9)	0.9 (0.1–2.7)	< 0.00001*

BCL2: B-cell lymphoma-2; CEA: Carcinoembryonic antigen; NEAT1: Nuclear paraspeckle assembly transcript 1; CA19-9: Cancer antigen 19−9; TGF-β1: Transforming growth factor beta-1; PC: Pancreatic cancer; Median (range) was employed to express the data. *: *P* < 0.05 indicating statistical significance.

### The diagnostic performance of the parameters investigated for PC

**Fig. 2:**
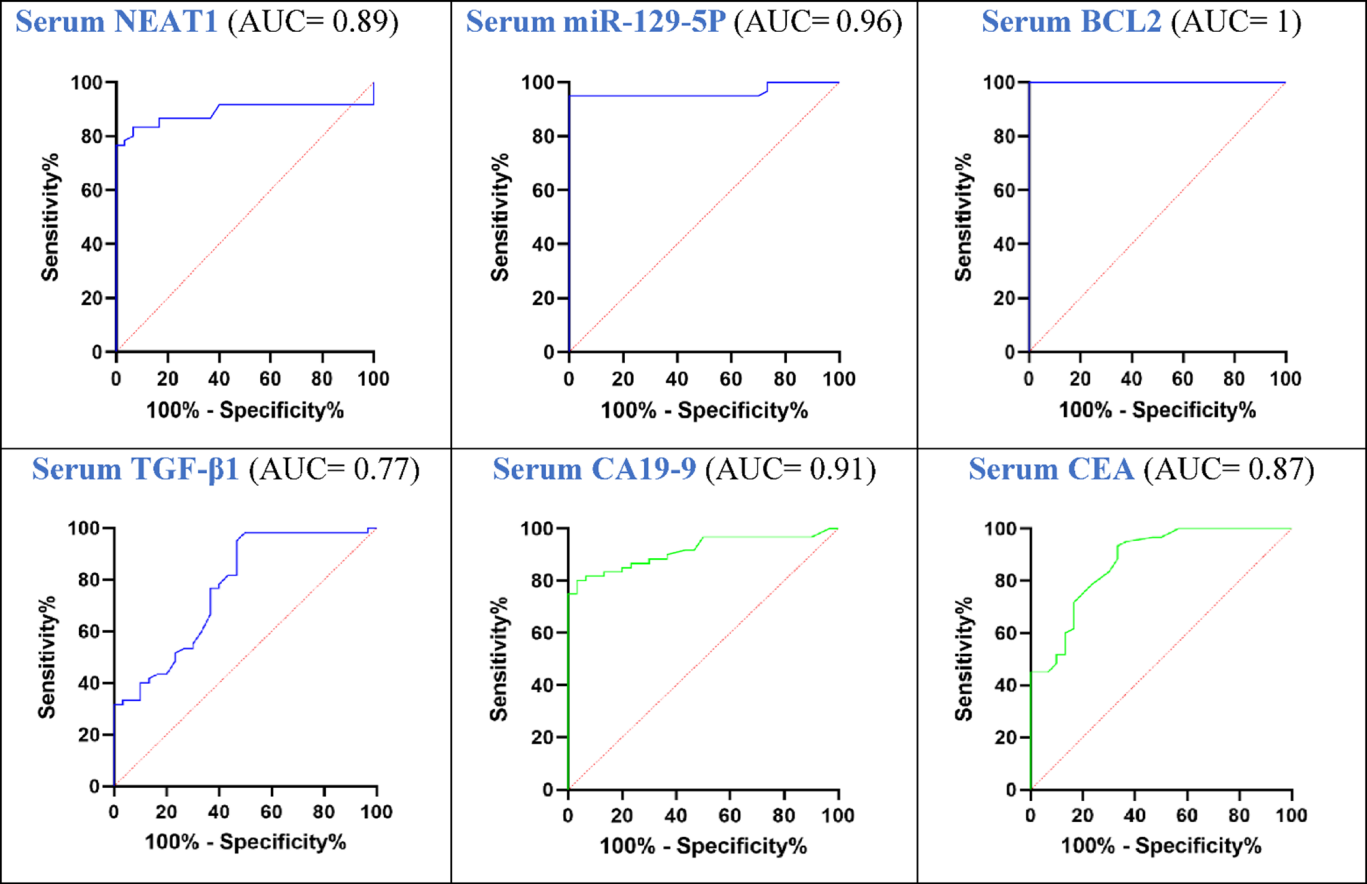
ROC curve for serum levels of NEAT1, miR-129-5p, BCL2, TGF-β1, CA19-9 and CEA as indicators for PC diagnosis. BCL2: B-cell lymphoma-2; CEA: Carcinoembryonic antigen; NEAT1: Nuclear paraspeckle assembly transcript 1; CA19-9: cancer antigen 19−9; TGF-β1: Transforming growth factor beta-1.

The diagnostic ability of the tested lncRNA NEAT1, miR-129-5p, and their related proteins such as BCL2 and TGF-β1 to differentiate between PC patients and control volunteers was analyzed utilizing the ROC curve **(**Fig. 2; Table 4**)**. Both lncRNA NEAT1 & miR-129-5p had powerful diagnostic performance for PC with specificity, sensitivity, and AUC of 93.3%, 83.3%, 0.89 at a cut-off value > 1.36 versus 100%, 95%, 0.96 at cut-off value ≤ 0.9, respectively. Additionally, BCL2 had the highest diagnostic accuracy for PC, with 1 AUC, 100% sensitivity, and 100% specificity at a cut-off value > 6.6 ng/ml. While TGF-β1 had the lowest diagnostic accuracy with 0.77 AUC, 76.7% sensitivity and 63.3% specificity at cut-off value > 46.3 ng/ml. On the other hand, sensitivity, specificity, and AUC for the classical PC tumor markers CA 19−9 and CEA were 80%. 96.7%, 0.91 at cut-off value > 26.5 U/ml and 75%, 80%, 0.87 at cut-off value > 1.65 ng/ml, respectively.

**Table 4:** The ROC curve data for discrimination between PC cases and controls.

Variables	Cut off	Sn (%)		Sp (%)	AUC	95% CI	*P*
NEAT1	> 1.36		83.3	93.3	0.89	0.82–0.96	< 0.00001*
miR-129-5p	≤ 0.9		95	100	0.96	0.92-1	< 0.00001*
BCL2 (ng/ml)	> 6.6		100	100	1	1–1	< 0.00001*
TGF-β1 (ng/ml)	> 46.3		76.7	63.3	0.77	0.66–0.88	0.00002*
CA19-9 (U/ml)	> 26.5		80	96.7	0.91	0.86–0.97	< 0.00001*
CEA (ng/ml)	> 1.65		75	80	0.87	0.80–0.95	< 0.00001*

AUC: Area under the curve; NEAT1: Nuclear paraspeckle assembly transcript 1; BCL2: B-cell lymphoma-2; CEA: Carcinoembryonic antigen; TGF-β1: Transforming growth factor beta-1 CA19-9: Cancer antigen 19−9; PC: Pancreatic cancer; Sn: Sensitivity; Sp: Specificity; CI: Confidence interval. *: *P* < 0.05 indicating statistical significance.

### Expression pattern of serum NEAT1/miR-129-5p/BCL2 and TGF-β1 in PC cases with different clinicopathological features

The PC cases were categorized using T staging in order to assess the association between serum levels of NEAT1, miR-129-5p, and their related targets BCL2 and TGF-β1 with cancer progression **(**Table 5**)**. In comparison to PC with the T2 stage, serum miR-129-5p level was reduced in those with T3 (median fold change 0.12; *P* = 0.03) &T4 stages (median fold change 0.12; *P* = 0.04) proving that the decline in serum miR-129-5p levels is an indication of cancer aggressiveness. Besides, serum BCL2 levels were significantly greater in PC cases with the T4 stage than in those with the T2 stage (*P* = 0.04), On the other hand, NEAT1 and TGF-β1 serum levels did not differ significantly between various T stages (*P* = 0.3, 0.49, respectively). Also, we attempted to recognize the connection between the serum levels of NEAT1, miR-129-5p, and their related targets BCL2 and TGF-β1 with lymphatic metastases in PC according to the conjecture that was previously mentioned. Based on their N-stage classification, the cases were analyzed for regional lymph node invasion (Table 5**)**. Expression of serum NEAT1, miR-129-5p, and their related targets BCL2 and TGF-β1 didn’t differ significantly among PC cases with different pN stages (*P* = 0.4, 0.71, 0.77, and 0.08, respectively).

Regarding the TNM stages comparison in PC cases **(**Table 5**)**, there were no notable variations in the serum NEAT1, miR-129-5p, or their associated targets BCL2 and TGF-β1. In order to determine whether these studied measures have a diagnostic value for metastasis, the PC patients were further divided into those that were in the curative (II and III) and metastatic (IV) stages and statistical findings displayed there were no significant differences regarding these parameters.

Additionally, statistical data showed that there were no notable differences in serum levels of NEAT1, miR-129-5p, and TGF-β1 between PC patients with different grades (*P* = 0.86, 0.92, and 0.52, respectively). However, a significant elevation in serum levels of BCL2 were observed in PC cases with grade 3 in contrast to those with grade 2 (*P* < 0.001).

**Table 5:** Serum NEAT1/miR-129-5p/BCL2 and TGF-β1 levels in PC cases with various clinicopathological features.

Clinicopathological features	Serum NEAT1 (fold change)	Serum miR-129-5p (fold change)	BCL2 (ng/ml)	TGF-β1 (ng/ml)
**pT stages**				
T2	3.3 (0.14–39.2)	0.22 (0.01–1.18)	19.4 (12.3–26)	48.5 (24-377.5)
T3	2.8 (1-39.8)	0.12 (0.01–0.43) ^a^	21.2 (10.6–26.5)	57.5 (41.5-337.5)
T4	2.9 (0.2–23)	0.12 (0.01–1.16) ^a^	23.1 (12.4–30.2) ^a^	52.5 (43.5-597.5)
*P*	0.3	0.043*	0.04*	0.49
**pN stages**				
N1	2.4 (0.2–23)	0.16 (0.01–0.79)	21.6 (15.4–26)	91.3 (44.5-597.5)
N2	3.1 (0.14–39.8)	0.15 (0.01–1.18)	21.2 (10.6–30.2)	48.5 (24-377.5)
*P*	0.4	0.71	0.77	0.08
**TNM stages**				
Stage II	2.4 (0.2–23)	0.17 (0.01–0.79)	21.6 (15.4–26)	91.3 (44.5-597.5)
Stage III	3.5 (0.14–39.8)	0.18 (0.01–1.18)	20.4 (12.3–30.2)	55.5 (24-377.5)
Stage IV	2.3 (0.5–5.9)	0.08 (0.03–0.47)	21.9 (10.6–26.4)	48 (41–235)
*P*	0.27	0.5	0.39	0.05
**Distant metastasis**				
Curative (II & III)	3.3 (0.14–39.8)	0.17 (0.01–1.18)	20.8 (12.3–30.2)	58.8 (24-597.5)
Metastatic (IV)	2.3 (0.5–5.9)	0.08 (0.03–0.47)	21.9 (10.6–26.4)	48 (41–235)
*P*	0.29	0.25	0.56	0.05
**Cancer grades**				
G2	3 (0.14–39.2)	0.14 (0.01–1.18)	20 (10.6–26.4)	52.5 (41.5-597.5)
G3	2.7 (0.2–39.8)	0.19 (0.01–0.56)	24.5 (12.4–30.2)	48.5 (24-337.5)
*P*	0.86	0.92	< 0.001*	0.52

BCL2: B-cell lymphoma-2; G: Grade; NEAT1: Nuclear paraspeckle assembly transcript 1; pN: Pathological node; pT: Pathological tumor; TNM: Tumor-node-metastasis; TGF-β1: Transforming growth factor beta-1; Data are expressed as median (range). *: A notable difference at *P* < 0.05 between the 2 or 3 groups. ^a^: A significant difference from PC cases with T2 stage at *P* < 0.05.

### Correlation between the investigated NcRNAs and their related targets BCL2 and TGF-β1 in PC cases

**Fig. 3:**
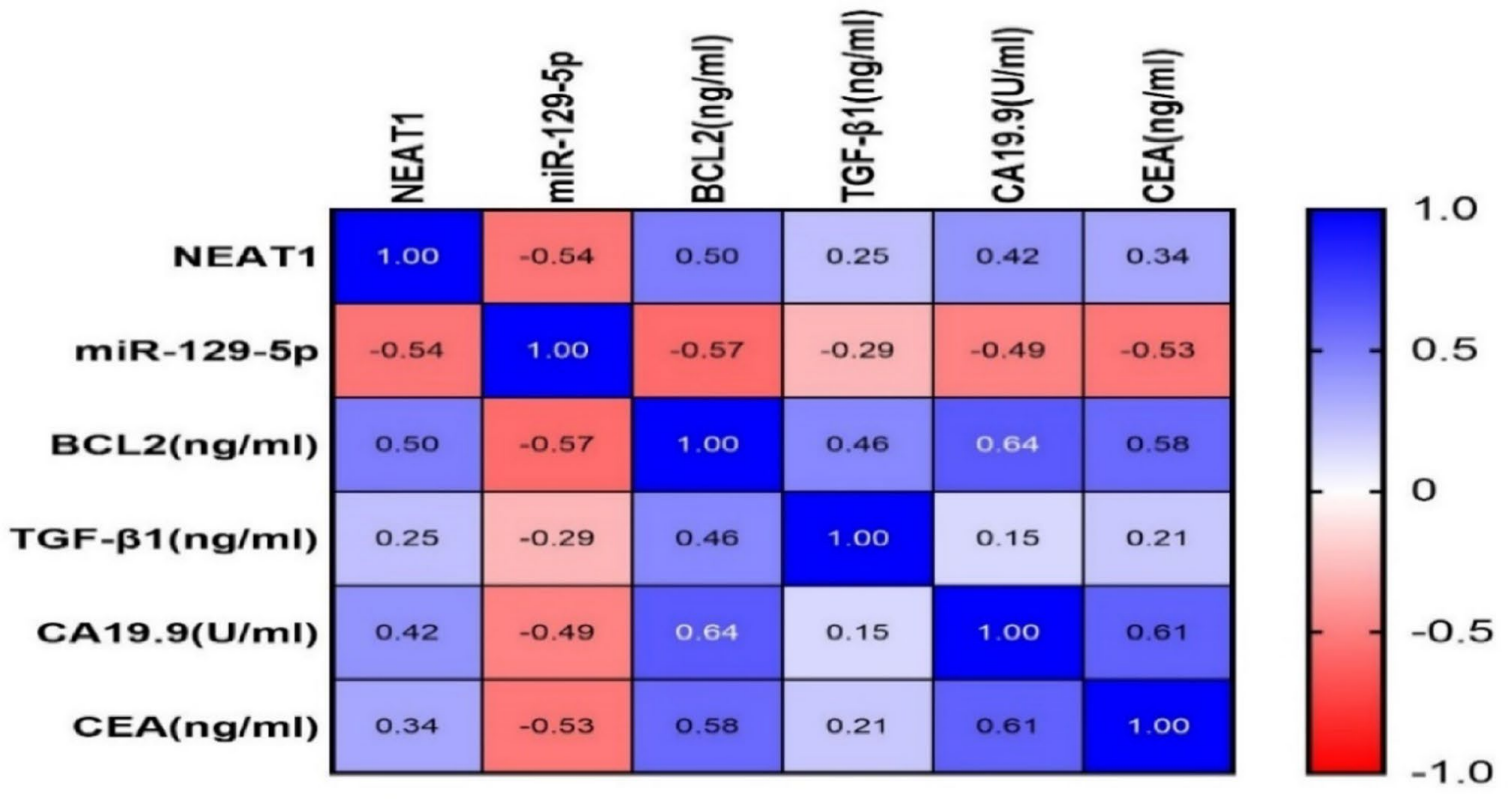
Spearman correlations among serum NEAT1, miR-129-5p and other investigated parameters in PC cases displayed on a relationship map. The map adopts a blue-red scale, where correlations close to 1 are displayed by blue, and correlations close to −1 are displayed by red.

Significant positive relationships were found between serum levels of lncRNA NEAT1 with BCL2 (*r* = 0.50, *p* < 0.001), TGF-β1 (*r* = 0.25, *p* = 0.02), CA19-9 (*r* = 0.42, *p* < 0.001) and CEA (*r* = 0.34, *p* = 0.001). On the other hand, a notable negative relationship was observed between serum levels of lncRNA NEAT1 and miR-129-5p (*r*= −0.54, *p* < 0.001). Also, there were marked negative correlations between serum levels of miR-129-5p with BCL2 (*r*= −0.57, *p* < 0.001), TGF-β1 (*r*= −0.29, *p*  =0.006), CA19-9 (*r*= −0.49, *p* < 0.001) and CEA (*r*= −0.53, *p* < 0.001). Moreover, serum BCL2 level was notably positively correlated with serum TGF-β1 (*r* = 0.46, *p* < 0.001) **(**Table 6; Fig. 3**)**.

**Table 6:** Correlation between serum levels of NEAT1, miR-129-5p and other parameters investigated in PC cases.

Variables	Correlation	NEAT1	miR-129-5p	BCL2	TGF-β1	CA19-9	CEA
**NEAT1**	R	1.00	−0.54	0.50	0.25	0.42	0.34
	*P*		< 0.001	< 0.001	0.02	< 0.001	0.001
**miR-129-5p**	R	−0.54	1.00	−0.57	−0.29	−0.49	−0.53
	*p*	< 0.001		< 0.001	0.006	< 0.001	< 0.001
**BCL2 (ng/ml)**	r	0.50	−0.57	1.00	0.46	0.64	0.58
	*p*	< 0.001	< 0.001		< 0.001	< 0.001	< 0.001
**TGF-β1 (ng/ml)**	r	0.25	−0.29	0.46	1.00	0.15	0.21
	*p*	0.02	0.006	< 0.001		0.16	0.04
**CA19-9 (U/ml)**	r	0.42	−0.49	0.64	0.15	1.00	0.61
	*p*	< 0.001	< 0.001	< 0.001	0.16		< 0.001
**CEA (ng/ml)**	r	0.34	−0.53	0.58	0.21	0.61	1.00
	*p*	0.001	< 0.001	< 0.001	0.04	< 0.001	

BCL2: B-cell lymphoma-2; CEA: Carcinoembryonic antigen; NEAT1: Nuclear paraspeckle assembly transcript 1; PC: Pancreatic cancer; TGF-β1: Transforming growth factor beta-1; CA19-9: Cancer antigen 19−9;. r: Correlation coefficient (range from − 1 to + 1), Statistically significant was adopted at *P* < 0.05.

### Bioinformatics analysis for detecting the correlation between NEAT1 and miR-129-5p

**Fig. 4:**
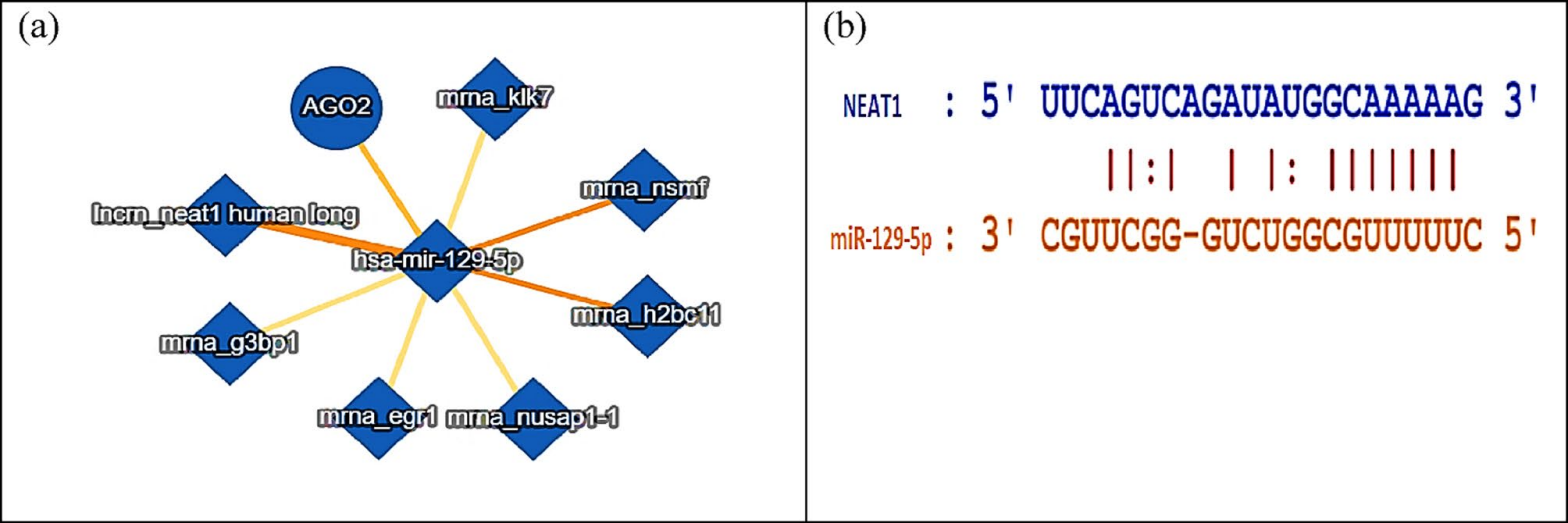
(**a**) miR-129-5p as a downstream target for NEAT1 using IntAct molecular interaction database, (**b**) The predicted binding sequence between NEAT1 and miR-129-5p based on ENCORI database.

**Fig. 5:**
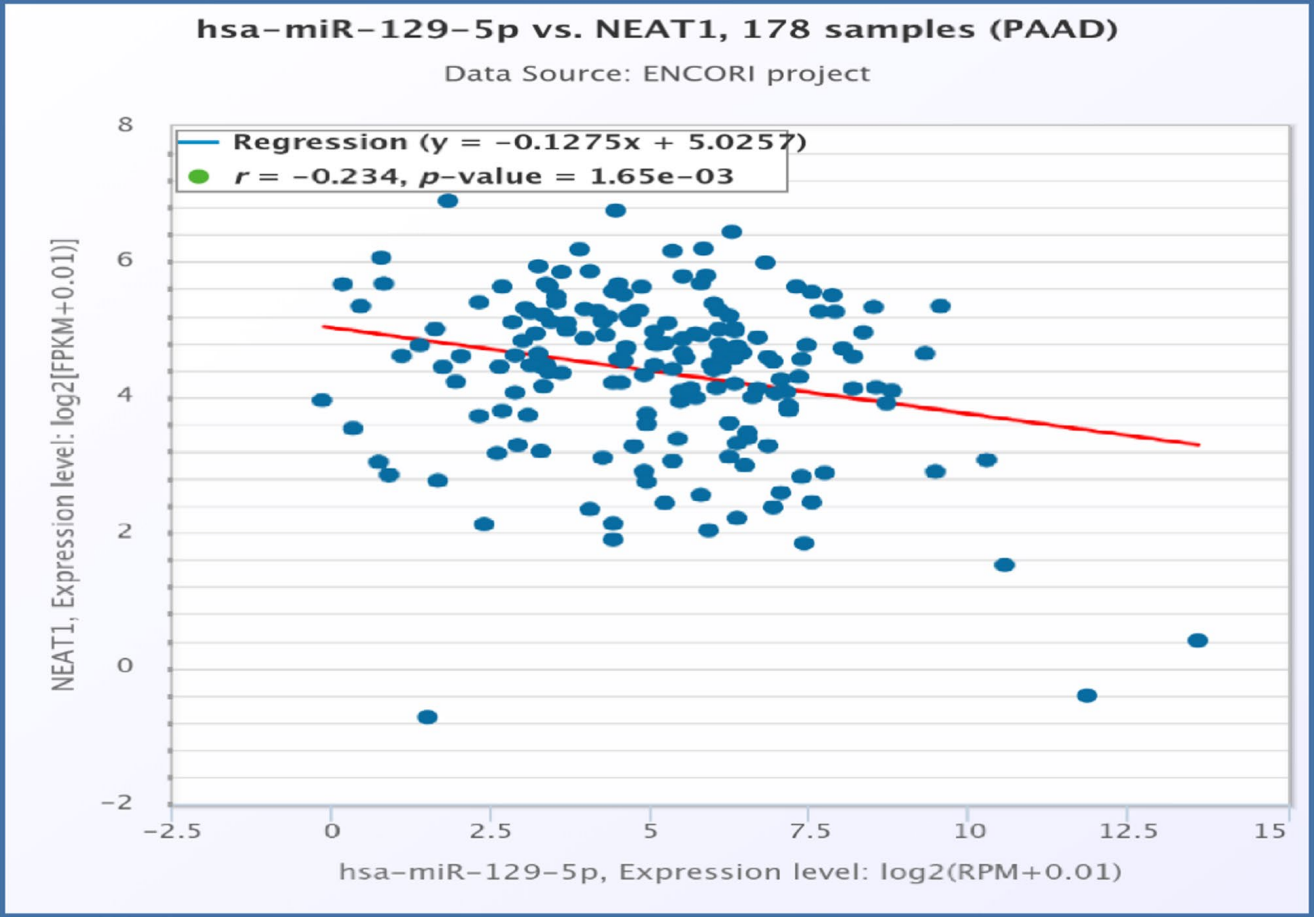
The correlation between expression levels of NEAT1 and miR-129-5p in the pancreatic cancer tissue regarding ENCORI database. PAAD: pancreatic adenocarcinoma.

IntAct molecular interaction database (accessed on 6th September 2024) was used as a bioinformatics tool to display the molecular interactions of miR-129-5p **(**Fig. 4a**).** The analysis elucidated a medium to high-confidence interaction between miR-129-5p and NEAT1 with MI score = 0.47. The binding sequence between miR-129-5p and NEAT1 was detected utilizing the ENCORI database (accessed on 6th September 2024) as illustrated in Fig. 4b. Moreover, a notable negative relationship between the expression levels of NEAT1 and miR-129-5p of pancreatic adenocarcinoma samples (*r*= −0.234) was found based on ENCORI database **(**Fig. 5**)**.

## Discussion

Pancreatic cancer is manifested by aggressive development and a late-stage diagnosis. Unfortunately, the exact pathophysiology of PC is still unclear^35,36^. Therefore, the early recognition and treatment of PC is crucial garnering lots of clinical and scientific attention^37^. However, the survival of PC patients can be significantly increased by about 5% above the usual rate by beginning life-saving therapy as soon as possible. Tumour tissues, particularly PC, are challenging to obtain. Nevertheless, the serological markers CEA and CA19-9, two of the current diagnostic techniques for PC, have poor sensitivity and specificity. The ncRNAs in plasma or serum have been the subject of numerous investigations as possible tumour indicators for PC diagnosis and prognosis^38,39^.

Based on our data, the current study is the first to investigate the relation between the serum levels of LncRNA NEAT1 and miR-129-5p in PC cases. In several cancer types, altered expression patterns of NEAT1 and miR-129-5p have been documented^40,41^. In PC, NEAT1 was reported to be highly expressed while miR-129-5p was reduced and both have lately garnered a lot of attention^42,43^. This study demonstrated that serum NEAT1 levels were markedly increased in PC cases compared to controls (median fold change 2.94) indicating that it plays a significant part in supporting oncogenesis. In harmony with our findings, several studies have demonstrated that NEAT1 expression was remarkably elevated in PC tissues and cell lines^12,23–25^.

Interestingly, in the present work, serum NEAT1 had a diagnostic capacity to differentiate between PC cases and controls at a cut-off value of > 1.36 with 0.89 AUC, 83.3% sensitivity, and 93.3% specificity. These results highlight the qualifications of serum NEAT1 levels to be considered as a tumour marker for PC diagnosis and suggest that NEAT1 may be an attractive target for therapeutic modulators. Similarly, NEAT1 was found to be a possible diagnostic indicator for breast cancer^44^ and hepatocellular carcinoma^45^with an AUC of 0.83, 0.98, sensitivity of 82%, 100%, and specificity of 80%, 88.9%, respectively. Moreover, NEAT1 showed an excellent discriminative ability for colorectal cancer (CRC) with AUC values of 0.907 and 0.947 reported by Wang et al.^46^ and Peng et al.^47, ^respectively. Additionally, a good discriminative ability for serum NEAT1 was reported between non-small cell lung cancer and control groups with AUC = 0.73^48^ and between prostatic cancer and controls with AUC = 0.7298^49^.

In spite of stabilizing some mRNAs and sponging some miRNAs, NEAT1’s secondary structure has the ability to bind to certain proteins and alter their molecular function or stability^50^. miR-129 has been regarded as a downstream target of NEAT1^15^. Our findings found that serum miR-129-5p levels were notably downregulated in PC patients compared to controls (median fold change 0.16) implying that it could serve as a tumor suppressor in PC development. This downregulation comes in accordance with several studies that reported that miR-129-5p level was repressed in PC tissues and cell lines^43,51^. Additionally, we revealed that serum miR-129-5p had the capacity to diagnose PC cases relative to controls at a cut-off value of ≤ 0.9 with 0.96 AUC, 95% sensitivity, and 100% specificity. That finding provides a novel treatment approach and an established diagnostic indicator for PC. Likewise, miR-129-5p has become an appealing diagnostic marker for hepatocellular carcinoma^45^ and breast cancer^52^ with an AUC of 0.997, 0.83, sensitivity of 100%, 69.44% and specificity of 97.2%, 83.33%, respectively.

Spearman correlation revealed a notable negative correlation between miR-129-5p & NEAT1 (*r*= −0.54, *p* < 0.001). Furthermore, bioinformatic analysis revealed that miR-129-5p could potentially interact and bind with NEAT1. This implies that NEAT1 may play a regulatory role in PC through sponging miR-129-5p. Likewise, the study by Shaker et al. found that the relative expression levels of serum NEAT1 and miR-129-5p had a strong negative relationship in HCC patients (*r*= −0.815)^45^. These findings are aligned with earlier studies that had proven NEAT1 as a negative regulator of miR-129-5p triggering the development of human clear-cell kidney cancer^27^ and the growth of hepatocellular carcinoma cells^26^. Additionally, NEAT1 was found to raise the miR-129 gene’s DNA methylation, which inhibits the tumour suppressor miR-129-5p expression in breast cancer^14^.

Increasing evidence indicates that NEAT1 is upregulated in various solid tumors, and its elevated expression is linked with unfavorable prognosis, poor survival, and tumor metastasis^53–56^. Besides, previous experimental studies documented NEAT1 as a possible prognostic biomarker of PC. Feng et al. revealed that upregulated NEAT1 was associated with TNM stage, tumor size, lymph node and distant metastasis, and poor prognosis^24^. Also, Huang et al. reported that poor survival and tumour progression were linked to elevated NEAT1 expression levels in PC patients^12^. In the present study, no notable relationship was obtained between NEAT1 expression levels and an aggressive clinical course in PC, however, the utilized sample type could be the origin of this discrepancy.

In harmony with Qiu et al. who revealed that the low miR-129-5p level in PC tissues was notably associated with T3- T4 pathologic tumor status, distant metastasis and not notably associated with lymph node status^43, ^our findings showed that serum miR-129-5p level was notably downregulated in PC cases with T3 or T4 stages in comparison to those with T2 stage & didn’t differ significantly among PC cases with different pN stages. Moreover, miR-129-5p serum level was reduced in metastatic stage PC cases than those in the curative stages. Additionally, several studies have regarded lower miR-129-5p as an appealing prognostic indicator for aggressive malignancies and a therapeutic target to repress the migration and spread of cancer cells^52,57^.

In accordance with the performed bioinformatic analysis that predicted anti-apoptotic BCL2 as a putative miR-129-5p downstream target, we also observed a significant negative relationship between them. These results are in harmony with an in vitro study by Qiu et al. who reported that miR-129-5p triggered apoptosis of PC cells by upregulating Bax, p21, cleaved caspase-3 and downregulating BCL2 protein^43^. Besides, miR-129 specifically downregulates BCL2 protein levels in nasopharyngeal cancer cells^52^. Moreover, NEAT1 indirectly modulated BCL2 expression in ovarian cancer cells by sponging miR-34a-5p^58^.

The present study revealed that serum BCL2 levels were notably increased in PC cases compared to controls with a diagnostic potential to discriminate PC with 1 AUC at a cut-off value > 6.6 ng/ml. Also, a higher serum BCL2 level was associated with advanced cancer grade and pathologic tumor status. These results come in line with other studies who reported that BCL2 was overexpressed in most of the PC tissue samples in contrast to the normal pancreas^59,60^. Similarly, according to Abu Siyam et al. study, serum BCL2 levels were higher in breast cancer cases than in healthy controls and clearly increased as the tumour’s grade developed^61^. Moreover, it was reported that circulating BCL2 in colorectal cancer (CRC) cases may represent the quantity of BCL2 expression in cancer tissue and could be effective as a predictive diagnostic in CRC^62^.

In the current study, TGF-β1 showed a notable negative correlation with miR-129-5p and a positive correlation with NEAT1. This finding comes in harmony with Zhang et al. study which reported that NEAT1 enhances pulmonary fibrosis by negatively modulating miR-9-5p that can control TGF-β1 signalling^19^. Moreover, miR-129-5p indirectly downregulates TGF-β1 in a number of cancer cell lines by targeting many angiogenesis-related genes^17,18^.

The multifaceted cytokine TGF-β1 can exhibit either proinflammatory or anti-inflammatory actions based on the cell niche^63^. TGF-β plays dual functions in the formation of tumors: either it suppresses tumors by inhibiting proliferation and inducing apoptosis as in the early stages of cancer, or it promotes angiogenesis and the invasiveness of tumor cells by modifying the immune system and the tumor microenvironment as in later stages^64–66^. According to our findings, serum TGF-β1 levels were considerably greater in PC cases than in healthy controls with the ability to diagnose and distinguish between them with 0.77 AUC, 76.7% sensitivity, and 63.3% specificity at cut-off value > 46.3 ng/ml. These findings are in accordance with Zhao et al. study which revealed that the sensitivity and specificity of TGF-β1 > 57.6 ng/mL for identifying PDAC cases were 83% and 76.4%, respectively. Also, they revealed that advanced tumour stage, lymph node metastasis, and distant metastases were linked with higher serum TGF-β1 levels^33^. However, our findings observed there was no notable relationship between its serum levels and poor prognosis of PC that could be explained by the variation of sample size.

The relationship between miR-129-5p and TGF-β1 has been clarified in some studies. According to Xiao et al. research, miR-129-5p is linked to renal fibrosis and reverses the consequences of TGF-β1-mediated EMT-related gene and protein expression through direct targeting of Smad interacting protein-1 (SIP1) and SOX4 expressions^67^. Moreover, miR-129-5p can function as a suppressor of gastric cancer development by suppressing SPOCK1, the downstream responsive component of TGF-β1 signalling pathways^68^. Additionally, it has been demonstrated that miR-129-5p inhibited TGF-β and α-SMA expression in the human kidney proximal tubular cell line HK-2^69^.

Interestingly, there are several emerging targets of miR‑129‑5p in PC, along with their functional significance. miR-129-5p overexpression in PC cell lines repressed migration, invasion, proliferation, and triggered apoptosis via downregulation of Pre–B-cell leukemia homeobox 3 (PBX3)^43^. Also, miR-129-5p has been established as a posttranscriptional modulator for importin 7 (IPO7). Its suppression prompted PC cells to increase the expression of IPO7 which induces PC cell growth and metastasis by repressing p53 and upregulating oncogenic lncRNA MALAT1^51^. Additionally, HMGB1 (High-Mobility Group Box 1) and ADAM9 (A Disintegrin and Metalloproteinase 9) are upregulated in PC and verified as targets for miR-129-5p across digestive malignancies implying more extensive anti-cancer actions and pending additional functional studies in PDAC^70,71^.

## Conclusion

The present study revealed that serum NEAT1, miR-129-5p, and BCL2 could be useful indicators for PC diagnosis. Also, low serum miR-129-5p was associated with advanced pathologic tumor status. Moreover, our results highlight and support the relationship between the serum levels of NEAT1, miR-129-5p, and related targets BCL2 & TGF-β1 and provide PC therapeutic targets. Further functional studies are recommended to verify that miR-129-5p targets BCL2 and TGF-β1 in PC.

## Supplementary Information

Below is the link to the electronic supplementary material.

Supplementary Material 1

## Data Availability

The datasets generated during and/or analyzed during the current study are available from the corresponding author on reasonable request.
