# Supplementary figures and images for: The diagnostic value of LncRNA NEAT1 targeting miR-129-5p in pancreatic cancer patients
Source: Sci Rep. 2025 Jul 29;15:27638. doi: 10.1038/s41598-025-12963-y (PMC12307695; doi:10.1038/s41598-025-12963-y)

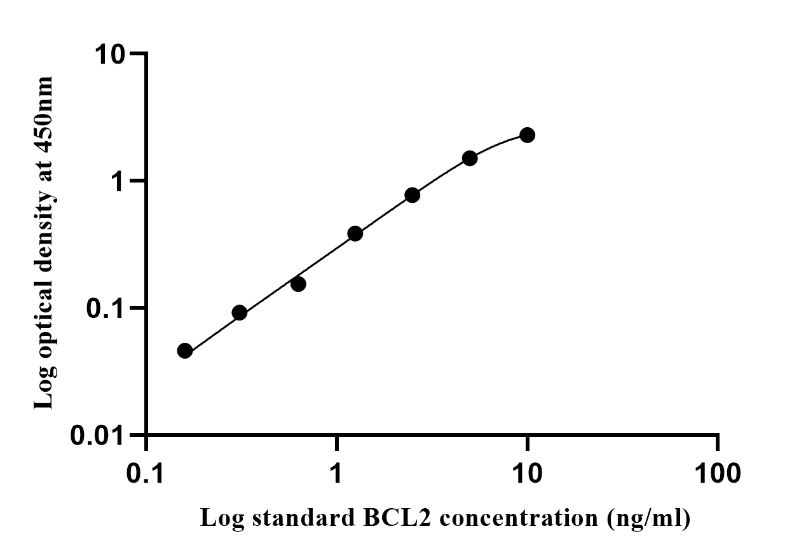


**Suppl. Fig. 1**. Standard curve for BCL2 (ng/ml)


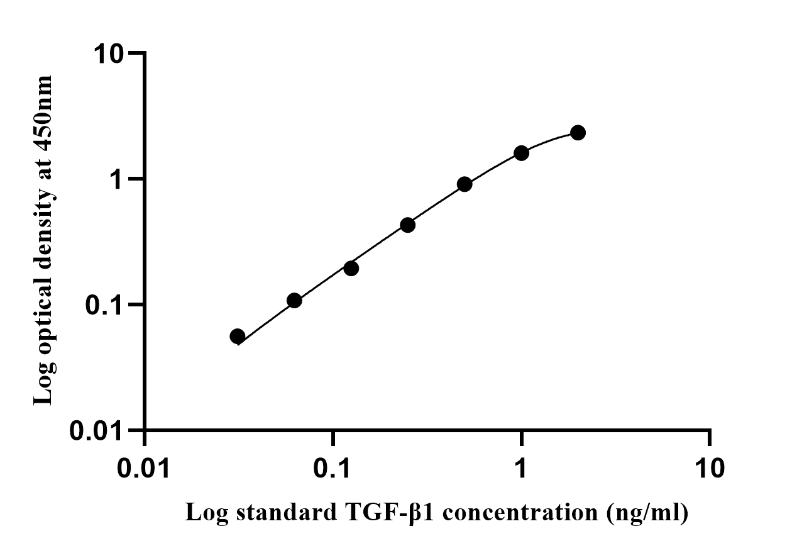


**Suppl. Fig. 2**. Standard curve for TGF-β1 (ng/ml)

Supplement: Supplementary file 1 — Supplementary Material 1 [file 41598_2025_12963_MOESM1_ESM.docx]
